# Anaemia, iron deficiency and inflammation prevalence in children in the Mount Cameroon area and the contribution of inflammatory cytokines on haemoglobin and ferritin concentrations: a cross sectional study

**DOI:** 10.1186/s40795-023-00748-3

**Published:** 2023-07-28

**Authors:** Sharon Odmia Sama, Germain Sotoing Taiwe, Rene Ning Teh, Gwendolyne Elobe Njume, Seraphine Njuontsop Chiamo, Irene Ule Ngole Sumbele

**Affiliations:** Department of Animal Biology and Conservation, University of Buea, Buea, Cameroon

**Keywords:** Anaemia, Iron defficiency, Inflammation, Children, Ferritin, Haemoglobin, Anaemia of inflammation

## Abstract

**Background:**

Iron deficiency (ID) and anaemia of inflammation (AI) coexist where infections and nutritional deficiencies are common. The aim of this study was to determine burden of ID, anaemia, inflammation and AI in children in malaria endemic Limbe, Mount Cameroon as well as decipher the contribution of some inflammatory cytokines on the concentration of haemoglobin and ferritin.

**Methods:**

A total of 520 children aged ≤ 15 years old from the Limbe Health District (LHD) were randomly selected and examined in a cross-sectional study for iron deficiency, anaemia, inflammation and inflammation anaemia. Collected blood samples were used for full blood count and inflammatory marker analyses with the aid of a haemoanalyzer and ELISA machine, respectively. Spearman’s rank correlation analysis was used to determine the correlation between cytokines and haemoglobin while multiple linear regression analysis was used to evaluate the effects of inflammatory cytokines on haemoglobin and ferritin concentrations.

**Results:**

The overall prevalence of anaemia, ID, IDA, inflammation and AI were respectively, 67.5%, 34.6%, 12.9%, 63.1% and 30.2%. Children aged 12‒15 years (P = 0.001), enrolled from the community (P < 0.001), whose parents are civil servants (P < 0.001), living in a home with 6‒10 occupants (P = 0.016), afebrile (P < 0.001) and malaria negative (P = 0.007) had the highest prevalence of ID while, children ≤ 5 years old (P = 0.001), with a family size of 1‒5 occupants (P = 0.033) had the highest prevalence of AI. Haemoglobin concentration positively correlated with concentrations of IFN-γ (P < 0.001), TNF-α (0.045) and ferritin (P < 0.001) while a negative correlation was observed with IL-10 (P = 0.003). In the multiple linear regression analysis only IL-6 significantly (P = 0.030) influenced haemoglobin concentration.

**Conclusions:**

While IL-6 is of significance in the pathology of anaemia, iron deficiency and anaemia of inflammation are of moderate public health concerns in the Mount Cameroon area. Hence, appropriate intervention against anaemia, ID and AI should be directed at children ≤ 5 years and counterparts > 10 years old that bear the highest burden.

**Supplementary Information:**

The online version contains supplementary material available at 10.1186/s40795-023-00748-3.

## Background

Iron deficiency (ID) is the primary cause of anaemia especially in low-income countries [1]. Over a billion people in all age groups suffer from it [2]. ID can be caused by inadequate intake of bioavailable iron or poor absorption of iron resulting from high intake of iron-inhibitory food or dietary fibres [3] or deficiency in other such micronutrients as folate and/vitamin B_12_, vitamin A [4], or genetic disorders. ID can be described as marginal when the production of iron-dependent proteins is compromised but haemoglobin levels are normal; mild when iron stores are depleted, and iron deficiency anaemia (IDA) when haemoglobin synthesis is reduced [5].

Anaemia resulting from iron deficiency is common among children and women especially in the developing countries [6]. Their deleterious effect on children both on the short-term and long terms have been well documented [7]. ID often goes unnoticed especially in patients who are not anaemic and/or do not complain of fatigue [8], thus, routine laboratory checks which requires measuring the ferritin concentration in blood is necessary [9]. Notwithstanding, ferritin is an acute phase protein (APP) highly influenced by inflammation and other infections [10], and its upshot on ID remains to be investigated in children resident in this malaria area of Mount Cameroon.

Inflammation is a protective innate immune response defence mechanism of the body whereby the immune system recognizes damaged cells, irritants, and pathogens [11]. Inflammation may not be detected if the infection is minor or if the immune system is apt in removing it (acute inflammation) thereby preventing disease. However, the changes that constitute inflammation are metabolically demanding and potentially destructive leading to conditions like cancer and diabetes (chronic inflammation) which in turn may lead to anaemia [12].

Anaemia ensuing from inflammation formerly called anaemia of chronic disease (ACD) is acute or chronic anaemia associated with conditions that cause inflammation including infections, cancer, autoimmune diseases, chronic kidney disease (CKD), and inflammatory bowel diseases (IBD) [10, 13, 14]. Although anaemia of inflammation (AI) is like ID in low serum iron (< 15 µg/L) [15], it is basically a disorder in iron distribution since macrophages of the liver, spleen and bone marrow responsible for recycling old red cells still retain their iron stores [16]. A person with AI thus will feel fatigued and intolerant to exercise [17] and consequently among children, it may affect their ability to study. AI coexists with ID in areas where nutritional deficiency and infections are common [18] and is considered the second cause of anaemia after IDA [19]. Up to 40% worldwide anaemia can be considered caused by AI or combined anaemia with AI contributing significantly [1, 20] and vary according to sex, age, geography and other disease prevalence [18]. The co-existence of ID and AI coupled with iron deficiency from malnutrition makes it difficult to get a specific diagnosis for ID [17] and therefore may lead to underestimation of the burden in a population.

In the Mount Cameroon area where diseases such as malaria and other infections abound, anaemia is almost inevitable. Previous studies conducted in this area have reported the prevalence and associated risk factors most often in relation to malaria [21–24], helminths [25, 26], HIV/AIDS [27, 28], infant feeding practices [29], nutritional status [30, 31] environmental factors [32] and urbanisation [33]. Yet, the contributions of iron deficiency and inflammation-linked anaemia- reported to be the major causes of anaemia to the overall burden and severity of anaemia have been infrequently studied. Therefore, the objective of this study was to determine the prevalence of iron deficiency, inflammation, and anaemia of inflammation as well as decipher the influence of some inflammatory markers on the concentration of haemoglobin and ferritin in children ≤ 15 years living in Limbe, in the Mount Cameroon area. Findings from the study will elucidate further the factors contributing to the pathogenesis of iron deficiency and anaemia in this at-risk group to facilitate proper management and control.

## Methods

### Study participants and site

This study was conducted in the Limbe Health District (LHD) in the Fako division of the Mount Cameroon area. The description of the town Limbe has been established in previous literature [21, 22]. The study participants included children ≤ 15 years old living in the area, whose parents consented to their participation in the study and who had not had blood transfused two months prior to the commencement of the study.

### Study design, sampling technique and unit

This was a cross sectional study conducted between December 2018 and August 2019. Upon receipt of administrative and ethical authorizations, participants were enrolled from their respective communities and hospitals within the LHD. Informed consent/assent forms were given to parents explaining the purpose, benefits, and risks of the study. Clinical evaluation was done after which structured questionnaires were administered to obtain socio-demographic data and clinical history. Blood samples were collected to determine the presence of malaria parasites, for full blood count (FBC) and biochemical analyses.

A convenience multistage method of sampling was used for data collection. For representativeness of each council, a health area was randomly selected from each of the 3 council areas that make up the LHD. This was followed by a random selection of representative health facilities and neighbourhoods in the selected health area. Following education by the community relay agents, enrolment into the study was consecutive in hospitals while in the community it was on planned visits. The formula *n = z*^*2*^*pq/d*^*2*^ [34] was used to calculate the sample size where *n* = required sample size; *z* = 1.96, the standard normal deviate for a 95% confidence interval (CI); *p* = 62%, the anaemia prevalence in the region [22]; *q* = (1-p) and *d* = 0.05, the acceptable error margin. The optimum sample size was 362. To allow for losses due to incomplete data entry the sample size was increased to 520 individuals.

### Data collection

Clinical examination for each child was done by a trained physician. Symptoms such as diarrhoea, headaches, and joint or muscle pains were recorded. The parent/guardian of the child was given a self-administered pre-tested questionnaire [S1 Questionnaire] developed for this study to be filled with the aid of an interviewer. This questionnaire included data on socio-demography and clinical symptoms. Axillary temperature was measured using a clinical thermometer and fever was defined as temperature ≥ 37.5 °C. Weight was measured using a Terraillon weighing scale to the nearest 0.1 kg while height was measured using a measuring tape to the nearest 0.1 cm. The anthropometric measurements were used to calculate nutritional indices: height-for-age (HA) for stunting, weight-for-age (WA) for underweight and weight-for-height (WH) for wasting, based on WHO growth reference curves [35]. A child was considered undernourished if he/she scored < -2 SD in one of the above indices [36].

### Laboratory methods

#### Determining malaria parasites

About 3 ml venous blood was collected using sterile techniques into labelled EDTA and dry tubes. The tubes were transported on ice to the Malaria Research Laboratory of the University of Buea for further analysis. Thin and thick blood films were prepared on the spot immediately after dispensing blood into tubes. The thin films were fixed with absolute methanol and with the thick films stained with 10% Giemsa stain for 15 min. They were examined for malaria parasites by microscopy according to standard procedures [37].

#### Haematology

Following the manufacturer’s instructions, the Nihon Kohden Celltac α (Tokyo, Japan) haemoanalyzer was used to run a full blood count analysis. Values for white blood cell (WBC) counts and haemoglobin (Hb) concentration were obtained. The classification of anaemia based on WHO [38] standard was as follows: Hb < 11.0 g/L for children 1–5 years, Hb < 11.5 g/dL for children 6 ‒ 11 years and Hb < 12.0 g/dL for children 12 ‒ 15 years old. Anaemia severity was categorised as mild anaemia = Hb 10.0 g/dL ‒ 10.9 g/dL for children 1 ‒ 5 years, Hb 11.0 g/dL ‒ 11.4 g/dL for children 6 ‒ 11 years and 11.0 ‒ 11.9 g/dL for 12 ‒ 15 years; moderate = 7.0 g/dL ‒ 9.9 g/dL for 1 ‒ 5 years, 7 ‒ 10.9 g/dL for 6 ‒ 15 years, and severe = Hb < 7.0 g/dL for all children.

#### Quantifying inflammatory cytokines and ferritin using ELISA

Blood in dry tubes were centrifuged at 3000 rpm for 5 min and the aliquots stored at – 20 °C until use. The inflammatory markers C-reactive protein (CRP), interleukin-1 beta (IL-1β), interleukin-6 (IL-6), interleukin-10 (IL-10), interferon-gamma (IFN-γ), tumour necrosis factor-alpha (TNF-α), and ferritin were measured using the sandwich ELISA technique with the ThermoFisher Scientific™ Multiskan™ Go Microplate Spectrophotometer (Waltham, Massachusetts, USA) ELISA machine following the manufacturer’s instructions.

The following cut-off values were used: CRP > 5 mg/L [39] confirmed the presence of any inflammatory process; IL-1β > 12 pg/mL [40], IL-6 > 50 pg/mL [41], IL-10 > 20 pg/mL [40], IFN-γ > 50 pg/mL [42] and TNF-α > 10 pg/mL [43]. Ferritin values < 12 µg/L for children 1 ‒ 5 years old and < 15 µg/L for children over five years old were used to define ID and IDA was defined as concurrent anaemia and low ferritin level [44] in the absence of inflammation. Anaemia of inflammation (AI) was defined as concurrent anaemia and inflammation without ID [45].

### Data analyses

Data was entered into Microsoft Excel 2016 and then exported to IBM-Statistical Package for Social Sciences version 21 (SPSS, Inc., Chicago, IL, USA) for statistical analysis. Graphs were plotted with R version 4.0.5 (Boston, Massachusetts, USA). Means and standard deviations were used to summarise continuous variables while categorical data was summarised as frequencies and percentages. Parasite density was log-transformed before analysis. Spearman’s rank correlation analysis was used to determine the correlation between cytokines and haemoglobin as well as ferritin. Multiple linear regression analysis was used to evaluate the effects of inflammatory cytokines on haemoglobin and ferritin concentrations. Significance was set at P-value < 0.05 at 95% confidence interval (CI).

### Ethical consideration

Clearance for the study with number 2018/811-05/UB/SG/IRB/FHS was obtained from the Ethical Review Board hosted by the Faculty of Health Sciences, University of Buea after obtaining administrative authorisation from the South West Regional Delegation of Public Health (R11/MINSANTE/SWR/RDPH/PS/430/940). Additionally, authorisations for community and hospital studies were obtained from the community chiefs and hospital director, respectively. Only participants who gave written consent documented by the investigator took part in the study. Children whose parents consented to the study were enrolled only after the purpose, risks and benefits of the study were clearly explained to them. It was emphasized that participation was fully voluntary and that a parent could at any time disallow his child to continue. All samples were coded to ensure confidentiality.

## Results

### Characteristic of the participants

The study included 520 participants, 46.5% (242) males and 53.5% (278) females. The mean (standard deviation: SD) age was 5.9 (4.2) years of which majority (56.3%, 293) were ≤ 5 years old. As shown in Table 1, most of the participants were enrolled from the hospitals (65.8%) and their parents had secondary level of education (42.9%). The prevalence of fever, malaria parasite, undernutrition, stunting, underweight and wasting in the study population were 36.9%, 37.9%, 18.2%, 16.0%, 4.7% and 6.8%, respectively. The geometric mean parasite density (GMPD)/µL of blood and prevalence of fever were significantly highest in children 1 ‒ 5 years old (928, 44%, respectively) when compared with their equivalents. Undernutrition prevalence varied significantly with age with children 11 ‒ 15 years old having the highest prevalence (22.2%) as well as in the prevalence of stunting (22.2%) as shown in Table 1.

**Table 1:** Socio-demographic and clinical characteristics of the study participants by sex and age

Parameter	Sex		Age group in years			Total
	Female	Male	1‒5	6‒10	11‒15	
**% (n)**	53.5 (278)	46.5 (242)	56.3 (293)	29.6 (154)	14.0 (73)	100 (520)
**Mean age (SD) in years**	6.1 (4.1)	5.6 (4.2)	2.7 (1.4)	8.3 (1.7)	13.3 (1.0)	5.9 (4.2)
**Mean (SD) Hb conc. in g/Dl**	10.5 (1.7)	10.5 (1.7)	10.3 (1.6)	10.5 (1.8)	11.1 (1.6)	10.5 (1.7)^ç^
***Socio-demographic factors***						
**Enrolment site**						
**Community (n)**	53.9 (96)	46.1 (82)	33.8 (69)	39.9 (71)	21.3 (38)	34.2 (178)
**Hospital (n)**	53.2 (182)	46.8 (160)	65.5 (224)	24.3 (83)	10.2 (35)	65.8 (342)
Education level of parent*						
**No formal/Primary (n)**	60.9 (84)	39.1 (54)	54.3 (75)	34.8 (48)	10.9 (15)	26.5 (138)
**Secondary (n)**	52.5 (117)	47.5 (106)	60.5 (135)	24.7 (55)	14.8 (33)	42.9 (223)
**Tertiary (n)**	48.4 (77)	51.6 (82)	52.2 (83)	32.1 (51)	15.7 (25)	30.6 (159)
***Clinical factors***						
**Mean (SD) temperature in °C**	37.4 (2.0)	37.3 (1.9)	37.5 (1.2)	37.2 (1.2)	37.2 (1.1)	37.4 (1.2)
**Malaria parasite prevalence (n)**	36.7 (102)	39.3 (95)	40.6 (119)	38.3 (59)	26.0 (19)	37.9 (197)^µ^
**GMPD (Range)/µL of blood**	703 (21,664)	634 (17,370)	928 (21,664)	442 (17,370)	318 (980)	669 (21,674)^a^
**Fever prevalence (n)**	36.3 (101)	37.6 (91)	44.0 (129)	31.2 (48)	20.5 (15)	36.9 (192)^b^
**Undernutrition prevalence (n)**	16.7 (46)	20.0 (47)	21.8 (62)	9.7 (15)	22.2 (16)	18.2 (93)^c^
**Prevalence of stunting(n)**	15.0 (41)	17.2 (40)	18.5 (52)	8.5 (13)	22.2 (16)	16.0 (81)^d^
**Prevalence of underweight (n)**	4.9 (11)	4.6 (9)	5.9 (17)	2.3 (3)	0.0 (0)	4.7 (20)^$^
**Prevalence of wasting (n)**	7.7 (11)	5.8 (8)	6.9 (19)	0.0 (0)	0.0 (0)	6.8 (19)^£^

* Education level of parent: No formal = not been to any school at all; Primary = 1–7 years of formal schooling; Secondary = 8–14 years of formal schooling; Tertiary = > 14 years of formal schooling

^ç^ Mean Hb concentration for gender and age groups respectively (P = 907 and P = 0.001)

GMPD: Geometric mean parasite density

^µ^ Difference in malaria parasite prevalence for gender and age group respectively (P = 0.547 and P = 0.071),

^a^ Difference in GMPD in the different age groups calculated using Kruskal-Wallis H test (P < 0.001)

^b^ Difference in febrile status in the different age groups (P < 0.001)

^c^ Evaluated for 510 participants and different for age groups (P = 0.005)

^d^ Calculated for 506 cases, difference in stunting in the different age groups (P = 0.007)

^$^ Underweight prevalence for gender and age group respectively (P = 0.877 and P = 0.255),

^**£**^ Wasting prevalence for gender and age group respectively (P = 0.527 and P = 0.638),

### Prevalence and severity of anaemia

The overall anaemia prevalence in the study population was 67.5% (351). Of the 351 anaemic cases 49.0% was mild, 45.0% moderate and 6.0% severe. A significantly higher prevalence of anaemia was found in participants enrolled from the community (81.5%, P < 0.001), children whose parent had no formal/primary education (73.9%, P = 0.018), were fishermen (81.7%, P = 0.012), who lived in a home with ≤ 5 persons (71.8%, P = 0.012) and were iron deficient (76.7%, P < 0.001) when compared with their respective counterparts. The prevalence was comparable with sex (P = 0.289), age (P = 0.443), fever (P = 0.095) and malaria status (P = 0.566). Although not significant, inflammation was higher in anaemic (69.5%) than non-anaemic (64.1%) children as indicated in Table 2.

The severity of anaemia was comparable with sex (P = 0.998), education level of parent (P = 0.699), family size (P = 0.551) and the presence of inflammation (P = 0.222). Anaemia severity however, varied significantly with age (P = 0.011), enrolment site (P = 0.012), parent’s occupation (P = 0.022), febrile state (P = 0.017), malaria parasite status (P = 0.001) and ID status (P < 0.001) as revealed in Table 2. The prevalence of mild anaemia was highest in the ≤ 5 years old (53.3%) while moderate anaemia prevalence was highest (66.7%) in their 12 ‒ 15 years old counterparts. While moderate anaemia was common (53.8%) in those within the community, mild and severe anaemia occurred commonly in children presenting to hospital (53.4%, 7.8%), respectively. Of significance, children who had fever and were malaria parasite positive had higher prevalence of mild (49.6%, 50.8%) and severe anaemia (10.7%, 11.5%), respectively, as opposed to those without. Children whose parents were jobless had the highest prevalence of mild anaemia (61.6%) while those whose parents were civil servants had the highest prevalence of moderate (55.0%) and severe anaemia (20.0%). A higher prevalence of moderate anaemia was observed in iron deficient children (57.2%) while those iron replete had a higher prevalence of severe anaemia (8.5%) as shown in Table 2.

**Table 2:** Prevalence and severity of anaemia by socio-demographic and clinical factors

Parameter	Category	Number	Anaemia prevalence % (n)	P-value	Severity of anaemia			P-value
					Mild	Moderate	Severe	
All		520	67.5 (351)		49.0 (172)	45.0 (158)	6.0 (21)	
Sex	Male	242	69.8 (169)	0.289	49.1 (83)	45.0 (76)	5.9 (10)	0.998
	Female	278	65.5 (182)		48.9 (89)	45.1 (82)	6.0 (11)	
Age group in years	1–5	293	66.6 (195)		53.3 (104)	39.5 (77)	7.2 (14)	**0.011**
	6–11	154	66.2 (102)	0.443	50.0 (51)	44.1 (45)	5.9 (6)	
	12–15	73	74.0 (54)		31.5 (17)	66.7 (36)	1.9 (1)	
Enrolment site	Community	178	81.5 (145)	**< 0.001**	42.8 (62)	53.8 (78)	3.4 (5)	**0.012**
	Hospital	342	60.2 (206)		53.4 (110)	38.8 (80)	7.8 (16)	
Education of parent	No formal/Primary	138	73.9 (102)		52.0 (53)	40.2 (41)	7.8 (8)	0.699
	Secondary	223	69.5 (155)	**0.018**	48.4 (75)	47.1 (73)	4.5 (7)	
	Tertiary	159	59.1 (94)		46.8 (44)	46.8 (44)	6.4 (6)	
Occupation	Farmer	141	62.4 (88)		47.7 (42)	48.9 (43)	3.4 (3)	**0.022**
	Civil servant	35	57.1 (20)		25.0 (5)	55.0 (11)	20.0 (4)	
	Others	120	63.3 (76)	**0.005**	42.1 (32)	51.3 (39)	6.6 (5)	
	Fishing	115	81.7 (94)		51.1 (48)	41.5 (39)	7.4 (7)	
	Jobless	109	67.0 (73)		61.6 (45)	35.6 (26)	2.7 (2)	
Family size	1–5	266	71.8 (191)		51.8 (99)	41.4 (79)	6.8 (13)	0.551
	6–10	233	64.8 (151)	**0.012**	45.0 (68)	49.7 (75)	5.3 (8)	
	> 10	21	42.9 (9)		55.6 (5)	44.4 (4)	0.0 (0)	
Fever	No	328	70.1 (230)	0.095	48.7 (112)	47.8 (110)	3.5 (8)	**0.017**
	Yes	192	63.0 (121)		49.6 (60)	39.7 (48)	10.7 (13)	
MP status	Negative	323	68.4 (221)	0.566	48.0 (106)	49.3 (109)	2.7 (6)	**0.001**
	Positive	197	66.0 (130)		50.8 (66)	37.7 (49)	11.5 (15)	
Inflammation	No	192	64.1 (123)	0.200	43.9 (54)	51.2 (63)	4.9 (6)	0.222
	Yes	328	69.5 (228)		51.8 (118)	41.7 (95)	6.6 (15)	
ID	No	340	62.6 (213)	**0.001**	54.5 (116)	37.1 (79)	8.5 (18)	**< 0.001**
	Yes	180	76.7 (138)		40.6 (56)	57.2 (79)	2.2 (3)	

MP: malaria parasite, ID: iron deficiency, P- values in bold are statistically significant

### Inflammation, ID, IDA and AI prevalence

The overall prevalence of inflammation (CRP > 5 mg/L), ID (age-related ferritin concentration < 12 µg/L and < 15 µg/L), IDA (concurrent ID and anaemia) and AI (concurrent anaemia and inflammation in the absence of ID) were respectively, 63.1%, 34.6%, 12.9% and 30.2%. Commonly, inflammation was highest in females (64.7%), children ≤ 5 years (65.5%), malaria parasite negatives (63.8%), those without fever (64.3%) and were not undernourished (63.3%) than their compeers. Of statistical significance, the prevalence of ID was highest in children 12 ‒ 15 years (56.2%, P < 0.001), those enrolled in the community (57.9%, P < 0.001), whose parents were civil servants (48.6%, P < 0.001), who lived in homes with 6‒10 occupants (41.2%, P = 0.016), had no fever (41.8%, P < 0.001) and were malaria parasite negative (39.0%, P = 0.007) when compared with their respective counterparts as revealed in Table 3.

Statistically significant differences in the prevalence of IDA were observed with age (P < 0.001), enrolment site (P < 0.001), parent’s occupation (P = 0.005), febrile status (P = 0.004). The 12–15 years old (23.3%), children within the community (22.5%), whose parents were fishermen (21.7%), and who were afebrile (16.2%) had the highest prevalence of IDA. In relation to AI, children 1 ‒ 5 years old and who lived in homes with 1 ‒ 5 occupants had the highest prevalence (36.9% and 35.3%) than their peers. This difference was significant at P = 0.001 and P = 0.033, respectively. Contrarily, children with fever (31.8%), malaria parasite positives (30.5%) and were undernourished (31.2%) had a higher preponderance of AI than counterparts that was none significant (Table 3).

**Table 3:** Prevalence of inflammation, ID, IDA and AI as affected by socio-demographic and clinical factors

Parameter	Category	Number examined	Inflammation prevalence % (n)	ID prevalence % (n)	IDA prevalence % (n)	AI prevalence % (n)
**All**		520	63.1 (328)	34.6 (180)	12.9 (67)	30.2 (157)
**Sex**	Male	242	61.2 (148)	36.0 (87)	13.6 (33)	29.3 (71)
	Female	278	64.7 (180)	33.5 (93)	12.2 (34)	30.9 (86)
	P- value		0.397	0.550	0.633	0.692
**Age group in years**	1–5	293	65.5 (192)	19.5 (57)	7.5 (22)	36.9 (108)
	6–11	154	59.7 (92)	53.2 (82)	18.2 (28)	20.1 (31)
	12–15	73	60.3 (44)	56.2 (41)	23.3 (17)	24.7 (18)
	P - value		0.419	**< 0.001**	**< 0.001**	**0.001**
**Enrolment site**	Community	178	62.4 (111)	57.9 (103)	22.5 (40)	27.0 (48)
	Hospital	342	63.5 (217)	22.5 (77)	7.9 (27)	31.9 (109)
	P - value		0.807	**< 0.001**	**< 0.001**	0.281
**Level of education**	No formal/Primary	138	66.7 (92)	37.7 (52)	15.2 (21)	32.6 (45)
	Secondary	223	61.0 (136)	33.6 (75)	14.8 (33)	33.2 (74)
	Tertiary	159	62.9 (100)	33.3 (53)	8.2 (13)	23.9 (38)
	P - value		0.553	0.676	0.103	0.116
**Occupation**	Farming	141	66.0 (93)	25.5 (36)	7.1 (10)	31.2 (44)
	Civil service	35	60.0 (21)	48.6 (17)	17.1 (6)	20.0 (7)
	Others	120	64.2 (77)	41.7 (50)	14.2 (17)	24.2 (29)
	Fishing	115	65.2 (75)	46.1 (53)	21.7 (25)	34.8 (40)
	Jobless	109	56.9 (62)	22.0 (24)	8.3 (9)	33.9 (37)
	P - value		0.604	**< 0.001**	**0.005**	0.220
**Family size**	1–5	266	60.2 (160)	28.7 (77)	12.0 (32)	35.3 (94)
	6–10	233	67.0 (156)	41.2 (96)	14.2 (33)	24.9 (58)
	> 10	21	57.1 (12)	33.3 (7)	9.5 (2)	23.8 (5)
	P- value		0.247	**0.016**	0.696	**0.033**
**Fever**	No	328	64.3 (211)	41.8 (137)	16.2 (53)	29.3 (96)
	Yes	192	60.9 (117)	22.4 (43)	7.3 (14)	31.8 (61)
	P - value		0.439	**< 0.001**	**0.004**	0.549
**Malaria status**	No	323	63.8 (206)	39.0 (126)	14.6 (47)	30.0 (97)
	Yes	197	61.9(122)	27.4 (54)	10.2 (20)	30.5 (60)
	P - value		0.672	**0.007**	0.146	0.918
**Malnutrition**	No	417	63.3(264)	34.3 (143)	12.7 (53)	30.0 (125)
	Yes	93	62.4 (58)	35.5 (33)	12.9 (12)	31.2 (29)
	P - value		0.865	0.827	0.960	0.819

P- values in bold are statistically significant

### Haemoglobin and ferritin concentrations and inflammatory markers

**Fig. 1:**
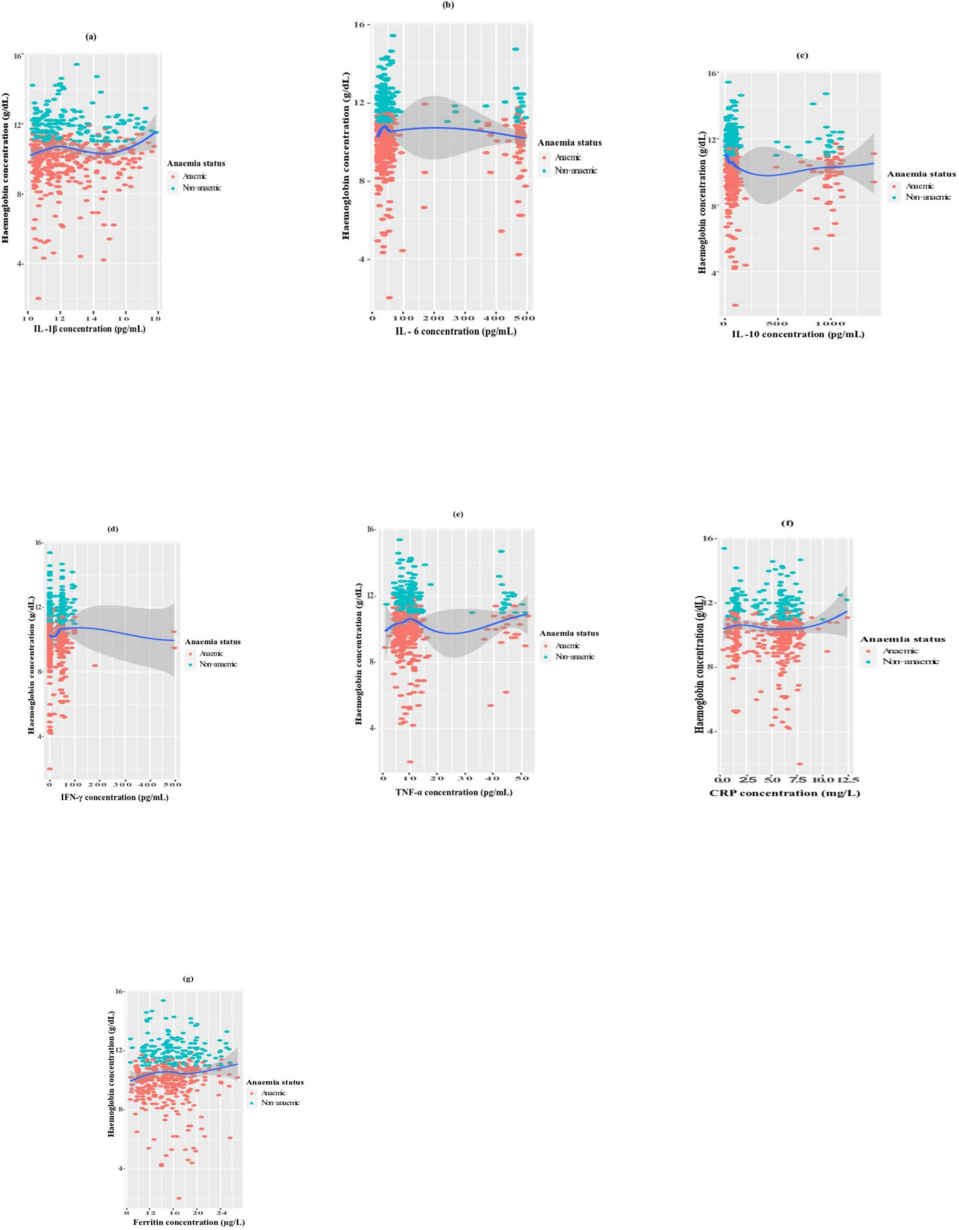
**(a)**-**(g)**: Correlations between haemoglobin concentration and some inflammatory markers

Correlations between haemoglobin and some inflammatory markers revealed a significant negative correlation between haemoglobin and IL-10 (P = 0.003) as shown in Fig. 1(c). Although not significant, a negative trend was also observed with haemoglobin concentration and IL-6 (P = 0.082) as revealed in Fig. 1(b). Meanwhile a significant positive correlation was observed between haemoglobin and IFN-γ (P < 0.001), TNF-α (P = 0.045) and ferritin concentration (P < 0.001) as shown in Fig. 1(d), (e) and (g), respectively. No significance however, was observed in the correlation between haemoglobin and IL-1β (Fig. 1(a) and CRP (Fig. 1(f) concentrations respectively.

**Fig. 2:**
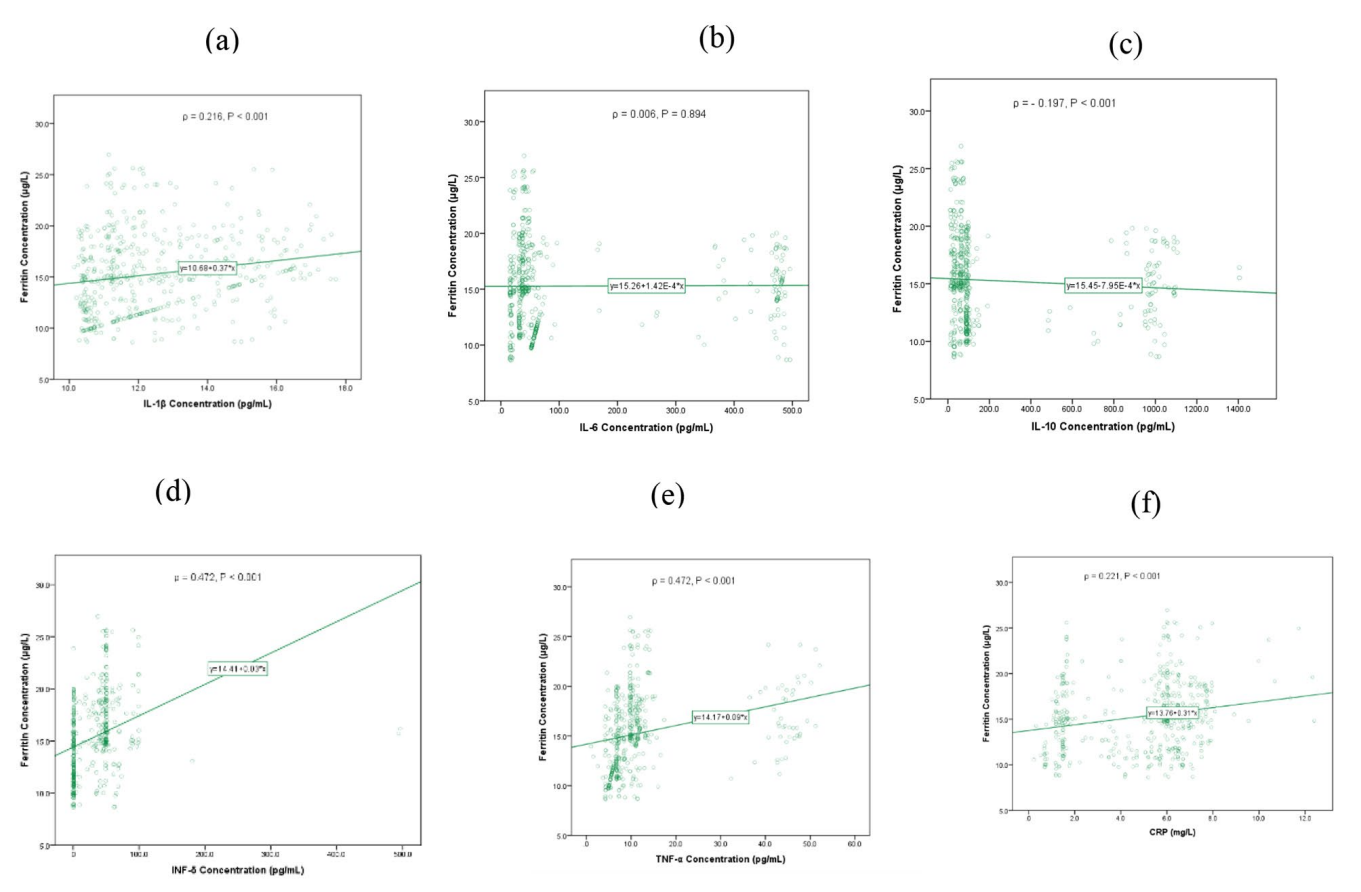
**(a)**-**(f)**: Correlations between ferritin concentration and some inflammatory Markers

On the other hand correlations between ferritin and some cytokines showed a significant positive correlation between ferritin and IL-1 (P < 0.001), IFN-γ (P < 0.001), TNF-α (P < 0.001) and CRP (P < 0.001) as shown in Fig. 2 (a), (d), (e) and (f), respectively while, a significant negative trend was observed between ferritin concentration and concentration of IL-10 (P < 0.001) (Fig. 2(c)). No significance was observed between ferritin concentration and IL-6 concentration as shown in Fig. 2(b).

### Influence of independent variables on haemoglobin and ferritin concentrations

Overall, the mean (SD) Hb was 10.5 (1.7) g/dL and mean (SD) ferritin was 15.28 (3.80) µg/L. Using a multiple linear regression model, the influence of socio-demographic and clinical factors as well as cytokine concentration on haemoglobin and ferritin concentrations were examined. Among the socio-demographic factors, the age (P < 0.001), enrolment site (P < 0.001) and family size (P = 0.007) had a significant positive influence on haemoglobin concentration as shown in Table 4. On the other hand, ferritin concentration was significantly influenced by sex (P = 0.011) and enrolment site (P < 0.001) and showed a positive correlation. Moreover, haemoglobin concentration showed no significant correlation with any of the clinical factors whereas ferritin concentrations was significantly influenced by febrile status (P = 0.013), malaria status (P = 0.040) and inflammation (P = 0.007). While ferritin correlated negatively with febrile and malaria status, respectively, it correlated positively with inflammation as shown in Table 4. Regarding the influence by inflammatory markers, haemoglobin negatively correlated (P = 0.026) with the concentration of IL-6. Meanwhile ferritin was significantly influenced by the concentration of IL-1β (P = 0.016), IL-6 (P = 0.044), IFN-γ (P < 0.001), TNF-α (P < 0.001) and CRP (P = 0.001) with all the associations being positive except for IL-6 which showed a negative trend (Table 4).

**Table 4:** Multiple linear regression analysis examining the influence of socio-demographic factors, clinical factors and inflammatory markers on haemoglobin and ferritin concentrations

Independent variable	Haemoglobin				Ferritin			
	Β	Standard Error (95% CI)	P-value	Model summary	Β	Standard Error (95% CI)	P-value	Model summary
***Socio-demographic factors***								
**Sex**	0.055	0.141 (-0.222–0.332)	0.697	R = 0.269	0.778	0.102 (0.176–1.381)	**0.011**	R = 0.419
**Age in years**	0.417	0.102 (0.217–0.616)	< 0.001*	R^2^ = 0.072	-0.268	0.221 (-0.702–0.166)	0.225	**R**^2^ **=** 0.176
**Enrolment site**	0.622	0.158 (0.312–0.932)	< 0.001*	AR^2^ = 0.061	3.198	0.343 (2.525–3.871)	< 0.001*	AR^2^ = 0.166
**Education level**	0.100	0.096 (-0.089–0.288)	0.300	F = 6.66	-0.197	0.208 (-0.606–0.213)	0.345	F = 18.230
**Occupation**	-0.025	0.047 (-0.118–0.068)	0.594	P < 0.001	-0.121	0.103 (-0.323–0.081)	0.241	P < 0.001
**Family size**	0.345	0.125 (0.099–0.591)	**0.006**		0.208	0.272 (-326–0.743)	0.444	
***Clinical factors***								
**Fever**	0.006	0.193 (-0.374–0.387)	0.974	R = 0.159	-1.134	0.455 (-2.029 - -0.238)	**0.013**	R = 0.295
**MP**	0.343	0.196 (-0.043–0.728)	0.081	R^2^ = 0.025	-0.949	0.461 (-1.856 - -0.042)	**0.040**	R^2^ = 0.087
**Inflammation**	0.005	0.200 (-0.388–0.398)	0.978	AR^2^ = -0.001	1.278	0.470 (0.353–2.203)	**0.007**	AR^2^ = -0.063
**Undernutrition**	-0.845	0.939 (-2.698–0.999)	0.366	F = 0.969	3.321	2.210 (-1.030–7.671)	0.134	F = 3.563
**Stunting**	0.619	0.901 (-1.156–2.394)	0.493	P = 0.454	-3.638	2.122 (-7.816–0.541)	0.088	P = 0.001
**Underweight**	-0.005	0.636 (-1.256–1.247)	0.994		-2.782	1.496 (-5.728–0,164)	0.064	
**Wasting**	0.557	0.461 (-0.350–1.464)	0.228		-1.565	1.084 (-3.700–0.570)	0.150	
***Inflammatory markers***								
**IL-1β/pg/mL**	0.065	0.052 (-0.037–0.167)	0.212	R = 0.157	0.265	0.109 (0.051–0.)	**0.016**	R = 0.431
**IL-6/pg/mL**	-0.002	0.001 (-0.004 – < 0.001)	**0.026**	R^2^ = 0.025	-0.004	0.002 (-0.008 - <0.001)	**0.044**	R^2^ = 0.186
**IL-10/pg/mL**	-0.001	< 0.001 (< 0.001–0.001)	0.471	AR^2^ = 0.013	-0.001	0.001 (-0.003 - <0.001)	0.096	AR^2^ = 0.176
**IFN-γ/pg/mL**	0.004	0.002 (< 0.001–0.008)	0.070	F = 2.152	0.022	0.004 (0.013–0.0.030)	< 0.001*	F = 19.476
**TNF-α/pg/mL**	0.009	0.008 (-0.005–0.024)	0.217	P = 0.046	0.066	0.016 (0.034–0.097)	< 0.001*	P < 0.001
**CRP/** mg/L	-0.029	0.033 (-0.093–0.035)	0.367		0.235	0.069 (0.100–0.370)	**0.001**	

P- value in bold are statistically significant, * statistically significant at P < 0.001

CI = confidence interval; AR^2^ = adjusted R^2^

## Discussion

Inflammation and iron deficiency are two major causes of anaemia in disease-endemic setting. A deficiency in iron supply leads to an imbalance in the homeostatic environment of the body spurring a series of acute phase responses and leading to inflammation. Inflammation leads to more iron retention [17], and then iron deficiency which further causes inflammation and subsequent anaemia. This vicious cycle of events leads to more iron retention and anaemia unless the root cause is diagnosed and treated. The study determined the prevalence of anaemia, iron deficiency and anaemia of inflammation in children in malaria endemic Limbe - Cameroon and how different social and clinical factors as well as inflammatory cytokines influence the concentration of haemoglobin and ferritin.

The overall anaemia prevalence of 67.5% is higher than the national prevalence of 62.5% recorded in children under five years [46], lower than the 77.7% obtained by Asoba et al. [29]. in the under-fives in some parts of the Mount Cameroon area, and comparable to 66.7% obtained in children in the Western Region of Cameroon [47]. This again confirms anaemia as a severe public health problem in the Mount Cameroon area hence, there is a dire need to re-strategize the current control methods being employed.

The overall iron deficiency prevalence (34.6%) is comparable to those obtained in Kenyan (36.9%) and Ugandan (36.5%) children [48], higher than 18.2% reported by Nazari [49] in Iranian children, and lower than 76.1% and 51.1% reported in children in India [50] and in a rural area in Cameroon [47], respectively. This high prevalence observed in Cameroonian children may be a result of poor feeding practices despite having a vast diversity of food as recently reported in a study in the same area [51]. In that study inadequate weekly consumption of meat and plantain (which are iron-rich) and fruits, (which facilitate iron absorption) were reported risk factors for anaemia, and iron-deficiency has been established as the main cause of anaemia [1]. Meanwhile iron deficiency anaemia prevalence (12.9%) was lower than the recorded 33.5% in children in the Gaza Strip [52]. This difference may be attributed to the different age strata used in the study. Whilst our study population included adolescents in both urban and semi-urban setting, those in Gaza were under-fives and lived in a setting already compromised by being marginalized.

Observation from the study revealed anaemia of inflammation prevalence (30.2%) is comparable to the ID prevalence (34.6%). This is in line with studies that say ID and AI often co-exist and together cause most of the anaemia encountered in disease-prone areas [1]. A plausible explanation is that during AI, iron absorption from the intestines is restricted leading to iron retention in the recticulo-endothelial system as ferritin thus causing ID [16]. Hence, the intensity of inflammation may be directly proportional to the amount of iron sequestered as observations from the study demonstrated a positive correlation between ferritin and the pro-inflammatory markers.

Sex-wise, findings from the study showed males had a higher prevalence of anaemia than females. Correspondingly, ID and IDA prevalence were higher in males than females. The high proportion of IDA in males than females is in line with Nazari et al. [49]. and Ewusie et al. [53]. in Ghana. This may be explained by the fact that the iron requirement for growth is higher in males than in females [54] and they gain more weight during their first years of life [55]. This may imply that ID is among the major contributors of the anaemia observed in this group. Conversely, AI was more prevalent in females, which is to be expected as they had a higher proportion of inflammation and therefore probably accounted for the observed anaemia than did ID. However, as a limitation, it is uncertain if the inflammatory process was acute or chronic since alpha 1-acid glycoprotein (AGP) which is more reliable in distinguishing past from present infection than CRP [56] was not assayed.

Findings from the study demonstrated children 12–15 years old had a higher occurrence of anaemia, ID and IDA. This is probably because there is a peak in iron requirements at adolescence resulting from expansion of red blood cell mass and growing muscle tissues [57]. Also, there is a possibility that adolescents tend to frequently snack and take carbonated drinks rather than eat proper meals or consume vegetables and fruits; these snacks are usually overly processed and may not contain the much-needed iron to meet their bodies’ demand.

In many African countries iron deficiency usually goes unnoticed except when diagnosed in the hospital in the cause of finding causes for another ailment [8]. This may account for the high prevalence within the community when compared with those enrolled at presentation in the hospital. Further observation demonstrated ID was more prevalent in children whose parents work in the civil service whereas anaemia and IDA was more common in children whose parents’ main occupation is fishing. Whilst being a civil servant may ensure a steady source of income, it is no guarantee that the quality of food consume by the children in these household is iron-rich or promote iron-absorption. Furthermore, in this conflict-hit area, food security is a challenge in many of such households offering hospitality to family members and other internally displaced individuals who have fled the violence in search for food, peace and security. This may have led to low dietary diversity as quantity will be preferred over quality. Moreover, the anaemia and IDA observed in children whose parent/caregiver were fishermen may be attributed to the deficiency in iron observed or more likely the result of blood loss due to some other infection than malaria as the negative trend observed between haemoglobin and malaria status in the regression model was not significant.

Relating to family size, children in homes with 6–10 members had a higher prevalence of ID than in homes with less than 5 members. This finding is in conformity with Psirropoulou et al. [58] in Greek children 1‒2 years old. A reasonable explanation may be that in large homes, iron intake may be reduced after reduction in food portions as food is spread more widely. Furthermore, these children may be more exposed to infections and other parasites. As a limitation in the study, the effects of other infections such as bacterial or helminths were not investigated, which would have divulged to what extent they contribute to the burden of ID.

ID and IDA observed in afebrile children may have resulted from inflammatory response to an infection which the immune system was trying to fight off as observations from this study revealed higher ID prevalence in malnourished and malaria negative children. In areas of high malaria transmission people develop some degree of immunity so, harbouring parasites without overt fever or malaria-linked symptoms is common [59]. Moreover, afebrile children may have been harbouring other blood-sucking parasites such as intestinal parasites which cause iron deficiency by direct blood loss resulting from intestinal bleeding, or appetite loss leading to reduced food intake or may prevent nutrient from being absorbed [60]. However, we did not assess intestinal parasites thus their role in iron deficiency in this study cannot be ascertained.

Congruent with previous studies [48, 61, 62,] malaria negative children had a higher prevalence of ID. The sequestration of iron in macrophages and liver cells in times of deficiency starves the malaria parasite [63] of iron thus serving as a protection against the disease in African children. It may also be that the production of nitric oxide, which has been shown to be detrimental against the malaria parasite, increases in ID states [64]. Hence, a probable increase in nitric oxide may be the cause of the high IDA burden seen in non-malarious children.

Both ID and IDA were common in undernourished than well fed children in accordance with Hagan et al. [65]. even though no significant difference was observed with ID. It is expected that undernourished children will be iron deficient and anaemic, as the association between iron status and malnutrition has been previously established [66]. The vicious cycle between malnutrition, iron deficiency and anaemia may lead to inflammation which will further exacerbate anaemia.

The significant negative correlation observed between IL-6 and haemoglobin concentration is in line with studies elsewhere [67]. IL-6 stimulates the production of hepcidin which is the main iron-regulatory hormone preventing absorption of iron from the intestines and release from macrophages leading to low iron levels which may result to anaemia. On another hand, being a pro-inflammatory cytokine, produced in an early response to TNF-α [68], IL-6 will lead to iron sequestration in the presence of inflammation or iron overload [67] by stimulating hepcidin to degrade ferroprotein, thereby reducing bone marrow supply of iron and thus decreasing serum iron concentration [69] leading to anaemia.

In line with Choucair et al. [70]., haemoglobin concentration correlated negatively with IL-10 concentration. IL-10 is an anti-inflammatory cytokine and acts to reduce inflammation by reducing the production of pro-inflammatory cytokines and free radicals such as nitric oxide [71]. A reduction in pro-inflammatory cytokines will lead to a decrease in inflammation and may lead to an increase in haemoglobin. IFN-γ is another pro-inflammatory cytokine which together with TNF-α, is produced in response to malaria infection [72]. Its production suppresses ferritin [73] starving the parasites of iron but also fostering ID and IDA [74] hence the positive correlation observed between haemoglobin and IFN-y levels.

Ferritin has been reported to correlate with inflammatory markers being on one hand a promoter and on the other a regulator of inflammation. The positive link between IL-1β, IL-6, IFN-y, TNF-α, CRP and ferritin shows that inflammatory markers can induce the expression of ferritin [75]. TNF-α and IL-1β have been shown to work in synergy with IL-6 to increase the production of more TNF-α and IL-1β, thus more CRP which in turn causes a corresponding increase in ferritin concentration [76]. This cascade of events usually results in iron retention in the liver and macrophages hence anaemia. Also, the presence of ferritin may stimulate the production of more of these cytokines including the anti-inflammatory IL-10 and multifunctional IL-6 which act to reduce the inflammatory response. This is observed in the negative trend between ferritin and IL-10.

Although the study had as limitations not assessing other haemoglobinopathies, nutritional deficiencies and behaviours that may have the potentials of influencing markers of iron deficiency and anaemia, nevertheless, the variables evaluated are critical in portraying the burden of anaemia, ID and the contributions of inflammatory cytokines to markers of anaemia, a severe public health burden in the region. There is a dire need for regular monitoring of these burdens to provide accurate data for the development of sustainable control strategies to alleviate the poor health of these children.

## Conclusions

With iron deficiency, iron deficiency anaemia and anaemia of inflammation as moderate public health concerns in children in the Limbe Health District of the Mount Cameroon area, along with its contribution to the overall burden of anaemia it is imperative to review the anaemia-control strategies in place. Children 12‒15 years had the highest prevalence of iron deficiency while those aged 1‒5 years old had the highest inflammation-anaemia prevalence hence, appropriate intervention should be directed at the groups with unreasonable burden. While iron deficiency contributes to about half the total anaemia prevalence, inflammation, mediated by cytokines and acute phase proteins, contributes just as much to the overall burden of anaemia. Therefore, the influence cytokines have on haemoglobin concentration should be considered in the management of anaemia.

## Electronic supplementary material

Below is the link to the electronic supplementary material.

Supplementary Material 1

## Data Availability

All datasets on which the conclusions of the research rely are presented in this paper. However, data is available from the corresponding author on reasonable request.
