# Supplementary material for: Anaemia, iron deficiency and inflammation prevalence in children in the Mount Cameroon area and the contribution of inflammatory cytokines on haemoglobin and ferritin concentrations: a cross sectional study
Source: BMC Nutr. 2023 Jul 28;9:94. doi: 10.1186/s40795-023-00748-3 (PMC10375674; doi:10.1186/s40795-023-00748-3)
Supplement: Supplementary file 1 — Supplementary Material 1 [file 40795_2023_748_MOESM1_ESM.docx]

**S1 QUESTIONNAIRE**

**DEMOGRAPHIC DATA**

1. Name …………………………………. Code /……. /
2. Age /……... / Sex /……/ Temperature /........../ Height /............... / Weight /............. /
3. Residence /……………… /
4. Region of origin /............................/
5. Religion 1 = Christian 2 = Muslim 3 = Naturalist 4 = Other

**SOCIO-ECONOMIC DATA**

1. Who is the head of your house? 1 = Father 2 = Mother 3 = Aunt 4 = Uncle 5 = Brother 6 = Sister 7 = Other 8 = specify /…………. /
2. How old is he/she? Age ranges 1 = ≤ 25, 2 = 26-35, 3 = 36 - 50, 4 = > 50
3. What level of education does the house head have? 1 = Primary 2 = Secondary 3 = University 4 = No education 5 = Other /…………. /
4. What is the family head’s occupation? 1 = Farmer 2 = Civil Servant 3 = Businessperson 4 = Private worker 5 = Fisherman 6= Jobless 7 = Retired
5. Marital status of house head 1= Married 2 = Divorced 3 = Widowed 4 = Common-law union 5 = Single
6. If married, type 1 = Monogamy 2 = Polygamy
7. How many children do you/your parents have? /…….../
8. How many people live in the house? / ….. /
9. In what material is the house built? 1= Plank 2 = Brick 3 = Other

**CLINICAL HISTORY**

1. History of bleeding? Yes / No **
2. Current malaria infection Yes / No
3. Current intestinal parasite infection? Yes / No

**KNOWLEDGE OF ANAEMIA**

1. Do you know about anaemia? 1 = Yes 2 = No
2. How can you tell a child has anaemia? 1 = Pale 2 = Weak 3 = Tired 4 = Other
3. Do you go to the doctor when you discover this? 1 = Yes 2 = No
4. If no, what do you do? 1 = Nothing 2 = Self-medicate
5. If you do self-medication, what do you use? 1 = Iron tablets 2 = Folic acid 3 = herbal medicine

**NUTRITIONAL STATUS**

1. How many meals does the family get per day? /…………/
2. How many meals do you get a day? /…………/
3. How often do you consume the following in a week: 1 = < 4 2 = 4 3 = > 4 Fruits..........b) Vegetables...........c) Meat............ d) Fish ......... e) Plantains...........
